# Comparison of COVID-19 Outcomes With Alpha-1 Antitrypsin Deficiency Prevalence in Europe: A Cross-Sectional Study

**DOI:** 10.7759/cureus.34293

**Published:** 2023-01-27

**Authors:** Yılmaz Sezgin, Sinan Becel, Aşkın K Kaplan

**Affiliations:** 1 Family Medicine, Istanbul Training Research Hospital, Istanbul, TUR; 2 Emergency Medicine, Bahçelievler State Hospital, Istanbul, TUR; 3 Family Medicine, Maltepe University Faculty of Medicine, Istanbul, TUR

**Keywords:** pandemia, europa, prevalence, alpha-1 antitrypsin deficiency, covid-19

## Abstract

Introduction

We hypothesized that the geographic distributions of COVID-19 and alpha-1 antitrypsin alleles prevalence are similar. We investigate whether there is a relationship between the geographical density of the COVID-19 pandemic and the distributions of alpha-1 antitrypsin alleles.

Methods

This research is a cross-sectional study. Alpha-1 antitrypsin PI*MS, PI*MZ, PI*SS, PI*SZ, and PI*ZZ genotypes frequencies of European countries were compared with the case and death data related to the COVID-19 pandemic as of March 1, 2022.

Results

A significant relationship was found between the rates of COVID-19 cases and the rates of individuals with alpha-1 antitrypsin PI*MS, PI*MZ, PI*SS, PI*SZ, and PI*ZZ genotypes allele in European countries.

Conclusions

The findings showed that the prevalence distribution of the alleles of the gene defect that causes alpha-1 antitrypsin insufficiency is related to the prevalence of COVID-19 pandemic data.

## Introduction

Alpha-1 antitrypsin (A1AT) belongs to the serpin protease inhibitor (serpin) family, as are alpha-1 antichymotrypsin, C1 inhibitor, antithrombin, and neuroserpin. It is known as alpha-1 antiproteinase and encoded as a proteinase inhibitor (PI) [1]. A1AT is synthesized by hepatocytes, macrophages, intestinal epithelial cells, and bronchial epithelial cells [2,3]. With a plasma half-life of five days, A1AT is found in all body fluids and most tissues. A1AT is an acute-phase protein and one of the potent regulators of neutrophil activation, acting through protease inhibition and other mechanisms [4]. On the other hand, A1AT is the most potent inhibitor of bacterial serine proteases, proteinase 3, and neutrophil elastase [4,5].

Most of the genotype distribution of A1AT in the human population are combinations of the M, S, and Z alleles. Furthermore, the PI*MM genotype is normal, encountered in 85-95% of the world population, and can synthesize 100% normal A1AT [6]. On the other hand, the genotype distributions of PI*MS, PI*MZ, PI*SS, PI*SZ, and PI*ZZ alleles are 5-15% worldwide. These alleles can achieve 80%, 60%, 55%, 40%, and 15% normal A1AT synthesis [7].

Most of the mutations in the A1AT gene result in mutant protein synthesis that lacks function and damages the cell where it accumulates [8]. The PI*ZZ genotype carries a high risk of diseases associated with A1AT insufficiency, while the PI*SZ, PI*SS, and PI*MZ genotypes are only potentially risky [9,10]. Especially the PI*SZ genotype causes pulmonary emphysema in smokers [7].

The SARS-CoV-2 virus (COVID-19) was identified as a result of research conducted on a group of patients with acute respiratory symptoms in the form of fever, cough, and shortness of breath in Wuhan Province, China, in late 2019 [11]. Approximately 81% of symptomatic patients infected with COVID-19 show mild, 14% severe, and 5% critical disease course [12].

A study covering 97 countries with a sample of 5.264 million projected that there are 190 million carriers of a risky allele in the A1AT. Of these alleles, 142 million (74.8%) are PI*MS, 42 million (22.3%) are PI*MZ, four million (2.1%) are PI*SS, 1.269 million (0.7%) are PI*SZ, 181,000 (0.1%) are PI*ZZ genotype [10].

We hypothesized that the geographic distributions of COVID-19 prevalence and A1AT allele prevalence are similar. Therefore, we aimed to investigate whether there is a relationship between the geographical density of the COVID-19 pandemic and the distributions of A1AT alleles in European countries.

This article was previously posted to the Authorea Preprint Repository on April 27, 2020.

## Materials and methods

This research is a cross-sectional study. No ethical approval was required for this study. The study data were obtained from public sources and related literature. All procedures performed in this study were by the ethical standards of the institutional and national research committee and with the 1964 Helsinki declaration and its later amendments or comparable ethical standards.

Variables 

Data [10] showing the A1AT PI*MS, PI*MZ, PI*SS, PI*SZ, and PI*ZZ genotypes numbers of European countries were compared with the case and death numbers related to the COVID-19 pandemic as published by Worldometer [13] on March 1, 2022. (Tables 1, 2).

**Table 1:** Distribution of the number of individuals with alpha-1 antitrypsin PI*MS, PI*MZ, PI*SS, PI*SZ, and PI*ZZ genotypes in European countries

Countries	Population	PI*MS	PI*MZ	PI*SS	PI*SZ	PI*ZZ
France	64057792	8901425	1443805	372279	120767	9794
UK	58255005	4795861	1496511	119127	73231	11503
Germany	82282988	3391943	1556160	37243	34173	7839
Italy	58090681	3378063	865226	53062	27182	3481
Netherlands	16783092	1006333	376051	17102	12782	2388
Belgium	10423493	1046086	322424	30705	18928	2917
Portugal	10735765	3067865	492100	367931	118036	4711
Spain	40548753	7418886	1232109	439616	146020	12125
Russia	139390205	1980058	1040348	7859	8437	2277
Switzerland	7623438	553018	105337	11232	4279	407
Denmark	5515575	290681	281904	4288	8317	4033
Estonia	1291170	31770	60514	213	810	772
Finland	5255068	76057	68696	283	511	231
Latvia	2217969	128034	184938	2166	6257	4519
Lithuania	3545319	115276	103232	1000	1790	802
Norway	4676305	221127	165348	2855	4270	1597
Poland	38463689	1113434	311919	8404	4709	660
Sweden	9074055	277523	265324	2273	4346	2077
Austria	8214160	352062	207626	4146	4890	1442
Greece	10749943	451874	41079	5162	938	43
Hungary	9880059	198126	56607	1023	584	83
Romania	22181287	241035	482069	677	2708	2708
Serbia	7344847	95131	183468	320	1235	1191

**Table 2:** Distribution of COVID-19 cases and deaths numbers in European countries

Countries	Population	Cases	Deaths
France	65455703	22702815	138367
UK	68310968	18886701	161361
Germany	84103285	14851512	123490
Italy	60355786	12782836	154767
Netherlands	17180071	6398114	21570
Belgium	11649737	3557314	30179
Portugal	10161242	3273624	21086
Spain	46776373	10977524	99410
Russia	146009080	16495369	352446
Switzerland	8730022	2806401	13234
Denmark	5816515	2651267	4649
Estonia	1327599	499083	2250
Finland	5550982	657445	2380
Latvia	1861299	662644	5256
Lithuania	2676924	906902	8434
Norway	5471737	1242160	1598
Poland	37797174	5680034	111586
Sweden	10174298	2448182	17207
Austria	9067632	2704530	14841
Greece	10362306	2421664	25860
Hungary	9631090	1789581	44051
Romania	19084536	2741945	63578
Serbia	8695382	1913861	15280

↵↵

Since the A1AT PI*MS, PI*MZ, PI*SS, PI*SZ, and PI*ZZ genotypes data of belongs other countries than Europe are accepted to be insufficient, and data on COVID-19 cases and deaths were not considered reliable for most of the countries in the Worldometer databased, only European countries data have been analyzed. In addition, the United Kingdom (UK) is regarded as the sum of England, Scotland, and North Ireland. A total of 23 countries, including France, the UK, Germany, Italy, The Netherlands, Belgium, Portugal, Spain, Russia, Switzerland, Denmark, Estonia, Finland, Latvia, Lithuania, Norway, Poland, Sweden, Austria, Greece, Hungary, Romania, and Serbia were included in the study.

The percentages of alleles were calculated by the number of alleles dividing the number of the total population in the article. The rates of cases and deaths were calculated by the number of cases and fatalities divided by the total population of the same date. This study evaluated the COVID-19 cases and deaths with Alpha-1 antitrypsin PI*MS, PI*MZ, PI*SS, PI*SZ, PI*SZ, and PI*ZZ genotypes rates in European countries.

Statistical analyzes 

All statistical analyzes were performed by using SPSS (IBM Corp. Released 2017. IBM SPSS Statistics for Windows, Version 25.0. Armonk, NY: IBM Corp). Whether it fits the normal distribution of data was assessed by The Kolmogorov-Smirnov test. The relationship between the data was evaluated using Spearman's Rho analysis. The threshold for statistical significance was taken as P<0.05.

## Results

According to Spearman's Rho correlation analysis results, there was a significant relationship between the rates of COVID-19 cases and the sum of A1AT alleles (PI*MS, PI*MZ, PI*SS, PI*SZ, and PI*ZZ) in European countries. (r_s_ = 0.706; p = 0.001) (Table 3). 

**Table 3:** Correlation between the rates of COVID-19 cases and deaths and alpha-1 antitrypsin alleles, PI*MS, PI*MZ, PI*SS, PI*SZ, and PI*ZZ in European countries. r_s_: Spearman's Rho; *Correlation is significant at the 0.05 level (two-tailed)

			Worldometer COVID-19 data	
			Cases rates	Death rates
Alleles of the alpha-1 antitrypsin gene	Sum of allele rates	r_s_	0.706	-0,175
		p	0.001*	0.425
		n	23	23
	PI*MS rates	r_s_	0.562	-0.138
		p	0.005*	0.529
		n	23	23
	PI*MZ rates	r_s_	0.680	-0.221
		p	0.001*	0.310
		n	23	23
	PI*SS rates	r_s_	0.574	-0.124
		p	0.004*	0.574
		n	23	23
	PI*SZ rates	r_s_	0.735	-0.219
		p	0.001*	0.315
		n	23	23
	PI*ZZ rates	r_s_	0.676	-0.226
		p	0.001*	0.299
		n	23	23
	PI*SZ and ZZ	r_s_	0.748	-0,225
		p	0.001*	0,301
		n	23	23

Similarly, according to Spearman's Rho correlation analysis results, there was a significant relationship between the rates of COVID-19 cases and A1AT PI*MS, PI*MZ, PI*SS, PI*SZ, and PI*ZZ genotypes alleles in European countries (r_s_ = 0.562, p = 0.005; r_s_ = 0.680, p = 0.001; r_s_ = 0.574, p = 0.004; r_s_ = 0.735, p = 0.001; r_s_ = 0.676, p = 0.001) (Table 3).

In addition, according to Spearman's Rho correlation analysis results, there was a significant relationship between the rates of COVID-19 cases and the sum of A1AT PI*SZ and PI*ZZ alleles in European countries (rs = 0.748, p = 0.001) (Table 3).

According to Spearman's Rho correlation analysis results, there was no significant relationship between the rates of COVID-19 death and the rates of individuals with A1AT alleles. 

## Discussion

Our study found a significant correlation between the sum of rates individuals with the PI*MS, PI*MZ, PI*SS, PI*SZ, and PI*ZZ alleles causing A1AT deficiency and the COVID-19 case rates. This finding supports our hypothesis that there is a relationship between COVID-19 and the distribution of genetic polymorphism of A1AT. As it is known, COVID-19 infection causes clinical disease in which important protease activities and coagulation mechanisms affect other systems, especially the respiratory system [14,15]. COVID-19 uses ACE2, cathepsins, endosomal cysteine ​​proteases, and the ligand Type 2 transmembrane serine protease (TMPRSS2), to enter cells [16,17]. Alpha-1 antitrypsin is one of the most critical molecules with antiprotease activity and inhibits neutrophil elastase activity in the lungs [4]. In addition, A1AT protects against proteolytic damage by inhibiting neutrophil elastase activity in the lungs [5]. It provides approximately 90% protection against elastolytic activity caused by elastase released from neutrophils in the lower respiratory tract [18,19]. A1AT indicates antithrombin III-like effects by inhibiting thrombin activity, such as heparin sulfate, and contributes to antiplasmin activity [20].

Very few people infected with the SARS-CoV-2 virus develop respiratory failure that requires mechanical ventilation. This clinical situation associated with high mortality shows a wide geographic variation [21]. In our study, a highly significant correlation has been found between the rates of COVID-19 cases in European countries and the rates of individuals with the A1AT PI*SZ genotype. Especially the A1AT PI*SZ genotype poses a high risk for A1AT failure and causes pulmonary emphysema in smokers [7]. In the European continent, the prevalence of the A1AT PI*SZ genotype is highest in the southern (1:483) and western (1:581) regions and lowest in the eastern part (1:11818) [7]. When the distribution of COVID-19 pandemics is investigated, Southern and Western European countries are more affected by the intensity of infection [13]. Additionally, mortality rates are high in this population [22]. A similar study conducted in the first months of the pandemic suggested that there may be a relationship between the geographic distribution of A1AT deficiency and COVID-19 deaths [23].

In a screening study conducted in Italy, 70 of 859 samples were found to have A1AT deficiency, and 80% had the PI*ZZ genotype [24]. Interestingly, more than 90% of the patients with this insufficiency are in the northern regions. It was also claimed that the proportion of both PI*S and PI*Z alleles of the A1AT gene was higher in the Northern Italy region [25]. Similarly, the current database of the COVID-19 pandemic shows that infection rates are higher in the Northern Italy region [26]. In addition, an Italy-focused study suggested that the distribution of the COVID-19 pandemic and A1AT deficiency coincided geographically, and this could not be explained by a random relationship [27]. In our study, a significant correlation was found between the rates of individuals with the PI*SS, PI*SZ, and PI*ZZ alleles causing A1AT deficiency and the COVID-19 case rates.

In a study conducted in the USA, it was claimed that the highest risk for A1AT deficiency was in whites, that is, those of European descent, followed by Mexicans and blacks, and the lowest risk was among those of Asian descent [28]. While the prevalence of the A1AT PI*ZZ allele globally is around 0.3%, it is estimated to be about 1% in the European continent [29]. In addition, almost half of the total 1.269 thousand PI*SZ genotypes calculated in the world are in Europe (74% in Spain, Portugal, France, and England), one-fifth in North and Central America (60% in the USA), and one-sixth in South America (55% in Brazil) [10]. In countries such as the USA and Brazil, especially in Southern and Western Europe, the case and death rates of the COVID-19 pandemic appear to be significantly higher [13]. It is noteworthy that recent studies are emphasizing the regional distribution relationship between COVID-19 and A1AT genetic deficiency [30]. In addition, there is a claim that A1AT deficiency may be responsible for the long COVID-19 process and that COVID-19 morbidity and mortality can be reduced by A1AT treatment [31].

Furthermore, the proportion of individuals with the PI*MM genotype synthesizing the normal A1AT protein varies between 85% and 95%, depending on the country [7]. Among alleles that cause A1AT insufficiency, the proportion of individuals with the PI*MS and PI*MZ genotypes considered low risk is 96%, and the rate of individuals with the PI*SS, PI*SZ, and PI*ZZ genotypes that are regarded as high-risk is around 4% [10]. Because 81% of symptomatic patients infected with COVID-19 have a mild, 14% have severe, and 5% have a critical illness, it is thought that the severity of the disease goes hand in hand with the A1AT allele rates [12]. In our study, the highest correlation was found between the sum of rates individuals with the PI*SZ and PI*ZZ alleles causing A1AT deficiency and the COVID-19 case rates.

In our study, a significant relationship was not found between the rates of COVID-19 death and the rates of individuals with Alpha-1 antitrypsin alleles. As it is known, many reasons, such as age, gender, chronic diseases, drug use, nutrition, and health care, play an active role in mortality rates. However, in light of all these, we think that this result in our study should not be accepted as a limitation for the validity of the hypothesis.

Limitations

Restriction of the source articles concerning A1AT data should be considered as our limitations.

## Conclusions

We conclude that the prevalence distribution of the alleles of the gene defect that causes A1AT insufficiency, which is one of the most crucial protective factors of lung tissue, is related to the prevalence of COVID-19 pandemic data. More detailed studies are needed on the relationship between A1AT and COVID-19 to confirm this argument, which we think can contribute significantly to combating the COVID-19 pandemic. Therefore, we recommend prioritizing studies that include genetic analysis in those with severe COVID-19 infection.
